# Five year trends in the serve size, energy, and sodium contents of New Zealand fast foods: 2012 to 2016

**DOI:** 10.1186/s12937-018-0373-7

**Published:** 2018-07-09

**Authors:** Helen Eyles, Yannan Jiang, Tony Blakely, Bruce Neal, Jennifer Crowley, Christine Cleghorn, Cliona Ni Mhurchu

**Affiliations:** 1 National Institute for Health Innovation, School of Population Health, The University of Auckland, Private Bag 92019, Auckland Mail Centre, Auckland, 1142 New Zealand; 2 Department of Public Health, University of Otago, Wellington, New Zealand; 3 The George Institute for Global Health, The University of Sydney, Sydney, Australia

**Keywords:** Fast foods, Portion size, Sodium, Energy intake

## Abstract

**Background:**

The nutritional composition of foods and beverages consumed away from the home has important implications for population health. Our objective was to determine if the serve size, energy, and sodium contents of fast foods sold at chain restaurants in New Zealand (NZ) changed between 2012 and 2016.

**Methods:**

Serve size and nutrient data were collected in annual cross-sectional surveys of all products sold at 10 major fast food chains. Changes over time may occur due to alterations in product availability or individual product reformulation. Linear regression adjusting for food group and chain was used to estimate overall changes in serve size and nutrients. Random effects mixed models were used to estimate reformulation changes on same products available for two or more years.

**Results:**

Across all products (*n* = 5468) increases were observed in mean serve size (+ 9 (3, 15) g, + 5%), energy density (+ 54 (27, 81) kJ/100 g, + 6%), energy per serve (+ 178 (125, 231) kJ, + 14%), and sodium per serve (+ 55 (24, 87) mg, + 12%). Sodium density did not change significantly. Four of 12 food groups (Desserts, Pizza, Sandwiches, and Salads) and four of 10 fast food chains (Domino’s, Hell Pizza, Pizza Hut, and Subway) displayed large, undesirable changes for three or more (of five) outcomes (≥10%; *p* < 0.05). One food group (Asian) and one chain (St Pierre’s) displayed large, desirable changes for two or more outcomes. The only significant reformulation change was a drop in sodium density (− 22 (− 36, − 8) mg/100 g, − 7%).

**Conclusions:**

The serve size and energy density of NZ fast food products has increased significantly over the past 5 years. Lower sodium concentration in new and reformulated products has been offset by overall increases in serve size. Continued monitoring and development and implementation of Government-led targets for serve size and nutrient content of new and existing fast food products are required.

**Electronic supplementary material:**

The online version of this article (10.1186/s12937-018-0373-7) contains supplementary material, which is available to authorized users.

## Background

Consumption of food prepared away from the home is increasing globally, and is an important contributor to population diets in many countries [1]. In the United States (US), food away from home as a share of household food expenditure has risen from ~ 26% in 1970 [2] to >50% in 2014 [3], with children consuming one quarter of their dietary energy from restaurant foods and beverages [4]. A similar trend is emerging in New Zealand (NZ) with food away from home contributing 25% in 2016 [5]. No recent data are available on the contribution food away from home makes to the energy intakes of New Zealanders, but in the most recent adult nutrition survey (2008/09) 14% reported consuming restaurant food and 28% reported consuming fast food over the past 24 h, with the highest rates observed for young adults (19 to 30 yrs.; 15 and 42%, respectively) [6]. Moreover, a recent (2017) market research panel survey reported 80% of NZ adults have consumed fast food in the past month, and 27% consumed fast food more than five times in that month [7].

Fast food can be defined as food which is generally cheap, requires minimal preparation and where no table service is provided [8]. The contribution that fast food makes to food away from home in NZ is currently unknown. However, fast food is of particular concern for population health because it is an independent predictor of body size [9]. Furthermore, compared with foods prepared at home, fast foods are generally more processed, higher in adverse nutrients such as sodium and saturated fat, and lower in positive nutrients such as fibre [9]. Serving sizes of foods and beverages available at fast food restaurants in the US have also increased steadily over time [10].

There is a clear mandate from the World Health Organization (WHO) to reduce serving sizes and population consumption of sugar and sodium [11–13]. As such, there is pressure for fast food companies to provide healthier options. Several countries have responded by implementing national sodium reduction strategies [14] and taxing foods and beverages high in sugar and sodium [15–17], and recently Public Health England outlined a series of actions to reduce children’s energy intakes, including portion size reduction [18]. However, the focus to date has been on packaged foods, and although existing nutrient profiling models exist which could be modified fast foods [19], there are currently no agreed serve size or nutrient guidelines for fast food companies to work towards. Moreover, the availability and composition of products available at popular global fast food chains varies markedly by country [20].

In NZ, there is currently no Government-led national sodium strategy or any food or beverage taxes aimed at improving health. However, there is a Government-led (voluntary) front-of-pack labelling system for packaged foods [21], and a national childhood obesity plan [22]; the obesity plan includes ‘The Healthy Kids Industry Pledge’ [23] where food companies have been encouraged (since 2015) to make voluntary pledges to improve their products and help reduce obesity rates for children. However, there are no product guidelines for industry; McDonalds is the only fast food chain to have made a pledge to date, and there are no specific, measureable goals to improve the serve size or nutrient content of their products. This is despite evidence that there is plenty of opportunity to improve the healthiness of NZ fast foods; a 2013 survey found burger combo meals sold at NZ fast food chains contribute up to 68% of the adult recommended dietary intake for sodium, and more than 94% of the WHO maximum recommended ideal free sugars intake [24].

The first objective of this project was to determine if the overall serving size, energy and sodium contents of fast foods sold by major NZ chain restaurants changed between 2012 and 2016, and whether any observed changes differed by food group or chain. This objective was assessed for all fast food products available for sale, where changes over time could arise due to new products having a different nutrient profile to discontinued products, and/or reformulation of products available for sale over time. The second objective more specifically examined reformulation within products available for sale in two or more years.

## Methods

### Data source

Annual cross-sectional surveys were undertaken between 2012 and 2016 of all food and beverage products available for sale at major fast food chains in NZ (≥20 stores nationwide). Fast food chains were defined as per Fleischhacker et al. [8] and described in the Background. The following data were collected by trained fieldworkers between February and March each year: product name, serving size, and nutrient information. It is not mandatory for any of this information to be available or displayed under the Australian and NZ Food Standards Code [25], and thus serving size and nutrient data were missing for some products. Data were recorded directly from company websites. Visits to one large store representing each fast food chain were also completed to capture any information not available on-line. Stores selected for visits were in Auckland, New Zealand’s largest city, and chosen based on size and location to provide the largest product range possible.

### Data management

Food and beverage data were entered into an Excel spreadsheet and classified using a three-tiered food classification system based on that developed by The Global Food Monitoring Group [26]. Identical products available for sale across years were matched manually using product name. Once matched, a unique code was allocated to each product to enable it to be tracked over time for reformulation analyses.

### Outcomes

There were five main outcomes of interest: serve size (g), energy density (per 100 g), sodium density (per 100 g), energy content per serve (kJ), and sodium content per serve (mg). Serve size was defined as the amount intended to be consumed in one sitting. For the majority of products the serve size was the same as the product unit or package size. However, for pizza and chicken products intended to be shared by a group, the serve size was as recommended by the manufacturer (*n* = 1539 and 115 total products, respectively).

### Preparation of the dataset for analysis

Additional file 1: Appendix 1 shows the preparation of the dataset for analysis. The initial dataset included 14,840 products. Three phases of data cleaning were completed: In phase 1 products with no recorded information on any outcome of interest were removed (*n* = 7281). In phase 2, duplicate products with different serving sizes were removed. For example, sides such as barbeque ribs were available in two sizes and thus only the largest serving size was retained (*n* = 137). Finally, in phase 3 all food groups and fast food chains with <100 products available for sale across all 5 years, and/or with data missing for one or more years were removed (*n* = 2091 products).

### Statistical analysis

The total number of product records included in each year were first summarised overall and by fast food group and chain separately. Descriptive information on serving size, energy, and sodium contents were presented for each year including the number of products with available information, mean, standard deviation, median and range. The sample was considered sufficiently large for the central limit theorem to apply, where the mean of the sample was considered approximately equal to the mean of the population [27].

For Objective 1, linear regression models were used to estimate the change in serve size, energy, and sodium contents with adjustment for fast food chain and food group to account for variation in the types of products sold during the study period. Overall analyses therefore estimated the change in nutrients over time, averaged across all food groups and fast food chains. Year was included as a continuous variable in the model (coded as 2012 = 0, 2013 = 0.25, 2014 = 0.5, 2015 = 0.75, and 2016 = 1) such that the coefficient gave an estimated change over 5 years. The interaction between year and fast food chain was tested in the main model to see if the observed change on nutrients was different between fast food chains. The same interaction was tested between year and food group. Where significant interaction was present, subgroup analyses were carried out for individual food groups and chains separately. However, results for sub-groups must be interpreted with caution due to multiple comparisons and diminishing statistical power. Average percentage change over the 5 years was calculated by dividing the adjusted mean change by an estimated mean for 2012 i.e. the overall mean minus half of the adjusted mean change. For completeness, all regression models were also run with year as a categorical variable to estimate the change between individual years e.g. 2012 to 2013, 2013 to 2014 etc.; these analyses are not included in the main results but are available in Additional file 1: Appendices 4 and 5.

For Objective 2 on reformulation analyses, random effects mixed models were used to estimate the 5-year change on serve size, energy and sodium contents for same products that are available for sale in two or more years. A random product effect was included in the regression model to account for within product change. Same covariates were considered in the fixed effects model as for Objective 1. Due to the limited number of products available for sale in two or more years, analyses by food group were only completed for outcomes where a significant change was observed over time.

All statistical analyses were performed using SAS version 9.4 (SAS Institute Inc., Cary, NC, USA). All statistical tests were two-sided at 5% significance level, with no adjustment for multiple testing.

## Results

### Characteristics of the data set

The total number of product records included for Objective 1 analysis was *N* = 5468 across 12 food groups and 10 major fast food chains (Additional file 1: Appendix 2). Overall product availability was relatively consistent across years (1359 in 2012, 1803 in 2013, 1211 in 2014, 1460 in 2015 and 1589 in 2016) but varied by food group and chain, ranging from *n* = 130 for Desserts to 1626 for Pizza, and from *n* = 173 for St Pierre’s (Sushi) to 1098 for McDonalds. The total number of unique products available for sale in two or more years and included in Objective 2 analysis was *N* = 1025 (Additional file 1: Appendix 3), including 632 products available in 2 years, 194 in 3 years, 107 in 4 years, and 92 available across all 5 years (a total of 2734 product records).

### Objective 1: change over 5 years for all products, and by food group and fast food chain

#### Serve size

**Fig. 1:**
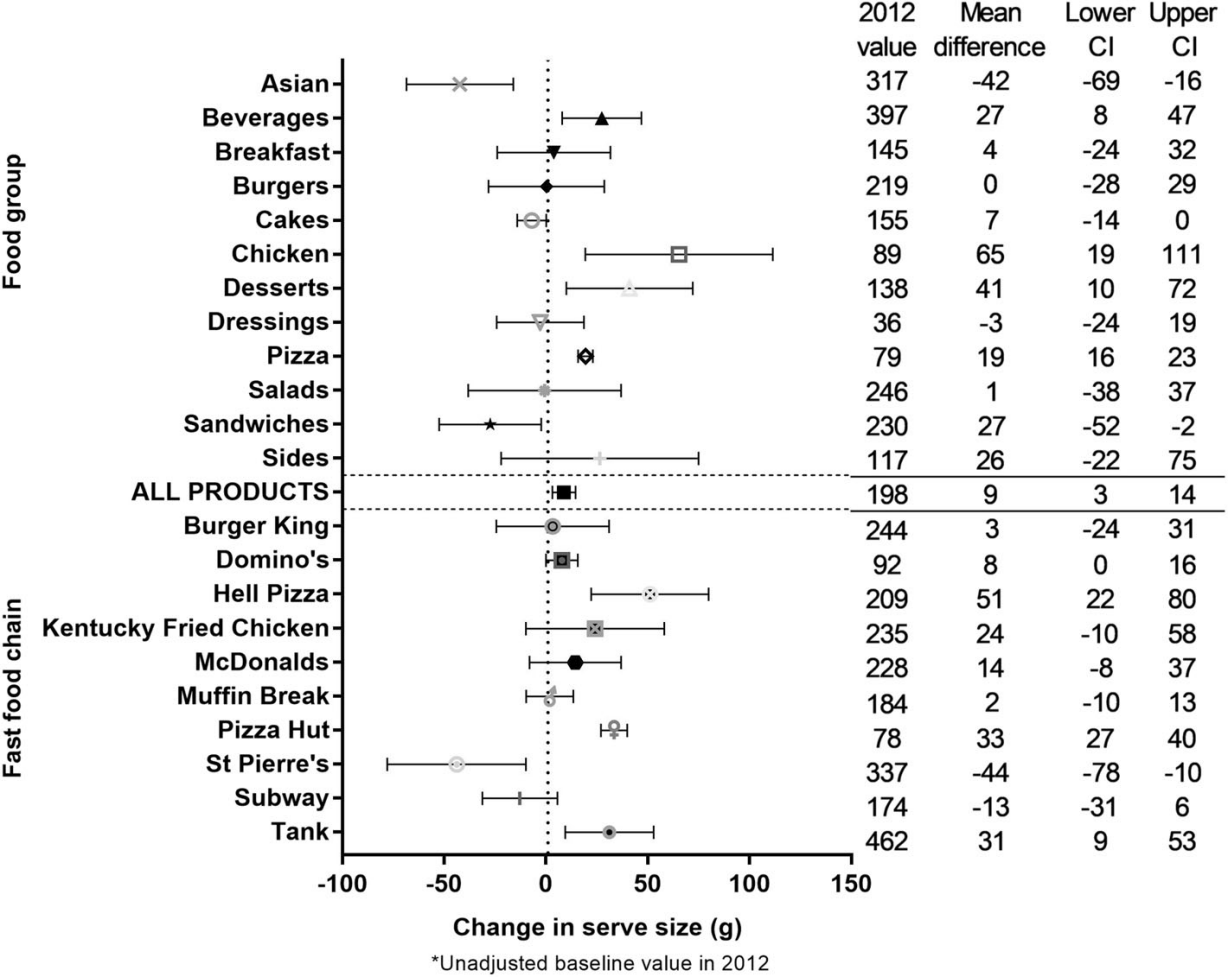
Five year changes in the serve size of fast food products from major NZ chains (2012 to 2016). * Unadjusted baseline value in 2012

The unadjusted mean (SD) serve size across all products in all years was 186.2 (135.8) g (Additional file 1: Appendix 2). The model-adjusted mean difference (95% CI) estimated over the 5 year period was + 8.8 (3.1 to 14.8) g, or equivalently an increase of 4.8% (Fig. 1). Significant interaction was found between year and food group, and between year and fast food chain (*p* < 0.0001). Large (≥10%), significant increases in serve size were observed for 3/11 food groups (Chicken (+ 70%), Desserts (+ 36%), and Pizza (+ 25%)), and 3/10 fast food chains (Domino’s (+ 10%), Hell Pizza (+ 34%) and Pizza Hut (+ 44%)). Large, significant decreases in serve size were observed for two food groups (Asian (Chinese food and Sushi; − 13%) and Sandwiches (− 11%)) and one fast food chain (St Pierre’s; − 14%) (Fig. 1).

#### Energy density

**Fig. 2:**
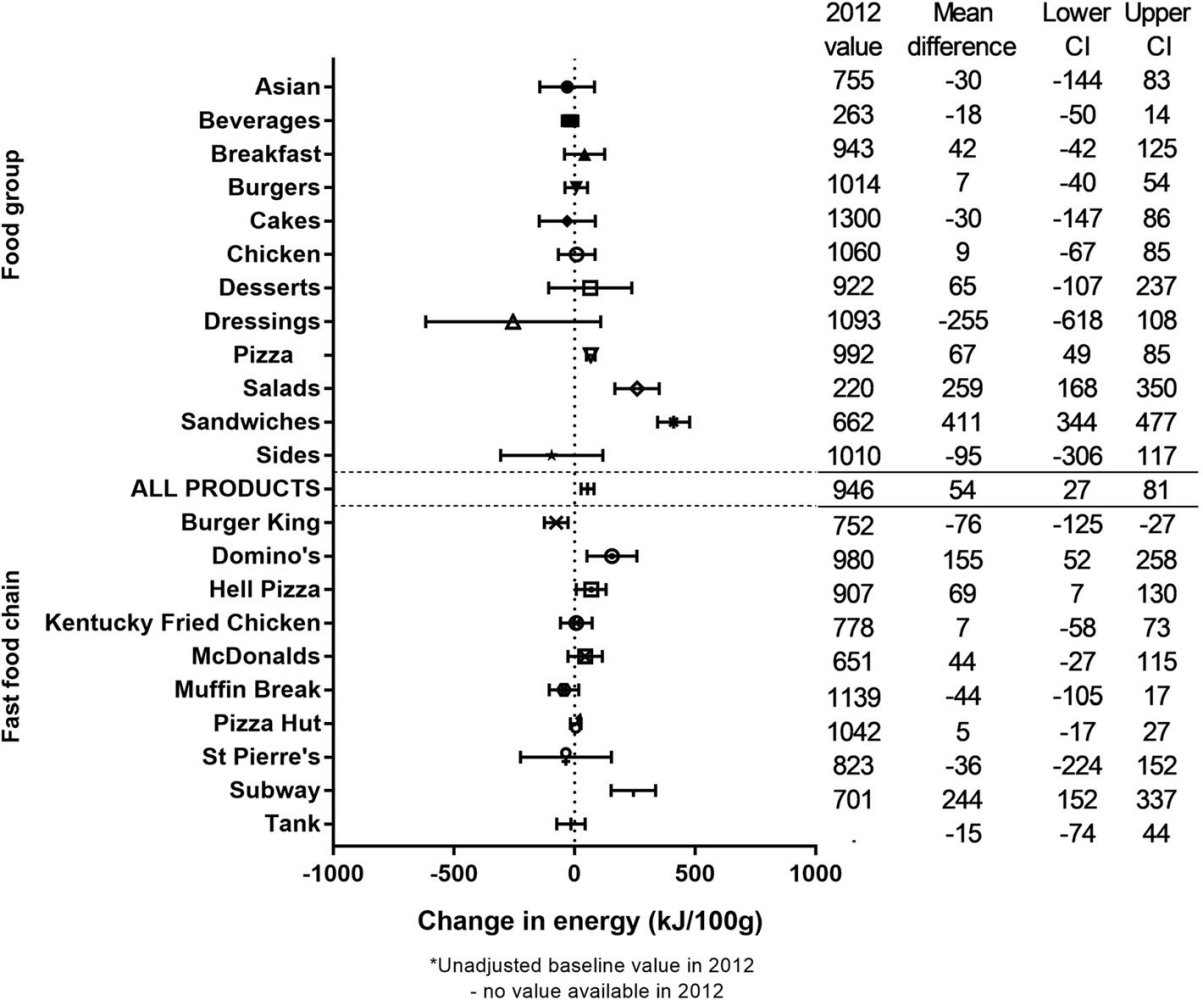
Five year changes in the energy density of fast food products from major NZ chains (2012 to 2016). * Unadjusted baseline value in 2012. - No value available in 2012

The mean (SD) energy density of all products over the 5 year period was 893.5 (484.6) kJ/100 g. There was a significant increase of + 54 (27 to 81) kJ/100 g predicted by the linear regression over the 5 years, or + 6% (Fig. 2). Significant interaction was found between year and food group, and between year and fast food chain (*p* < 0.0001). One food group (Sandwiches) showed a large, significant increase in energy density (+ 78%), but no food groups showed large (i.e. 10% or more) significant decreases. Across fast food chains one showed a large, significant increase in energy density (Domino’s Pizza; + 15%), and none showed large, significant decreases (Fig. 2).

#### Sodium density

**Fig. 3:**
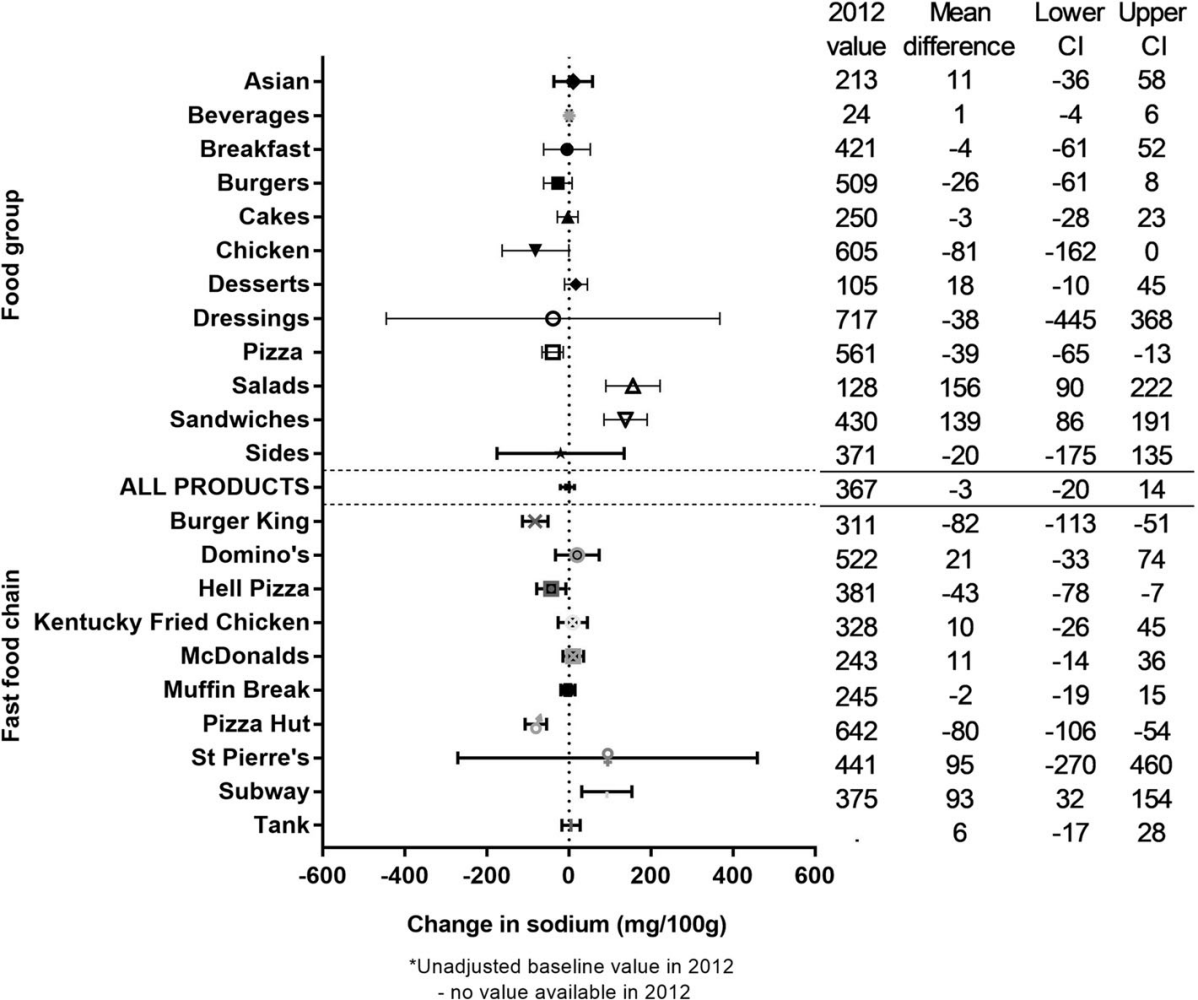
Five year changes in the sodium  density of fast food products from major NZ chains (2012 to 2016). * Unadjusted baseline value in 2012. - No value available in 2012

The mean (SD) sodium density across all products over the 5 years was 352.6 (316.6) mg/100 g. Overall, there was no significant change observed over the 5 year period (− 3.2 (− 20.4 to 14.1) mg/100 g; Fig. 3). However, significant interaction was found between year and food group, between year and fast food chain (*p* < 0.0001). Large, significant increases in sodium density were observed for Salads and Sandwiches (+ 102% and + 39%, respectively), and for Subway (27%). Large, significant decreases in sodium density were also observed for three fast food chains (Burger King (− 23%), Hell Pizza (− 12%) and Pizza Hut (− 13%), but no food groups.

#### Energy per serve

**Fig. 4:**
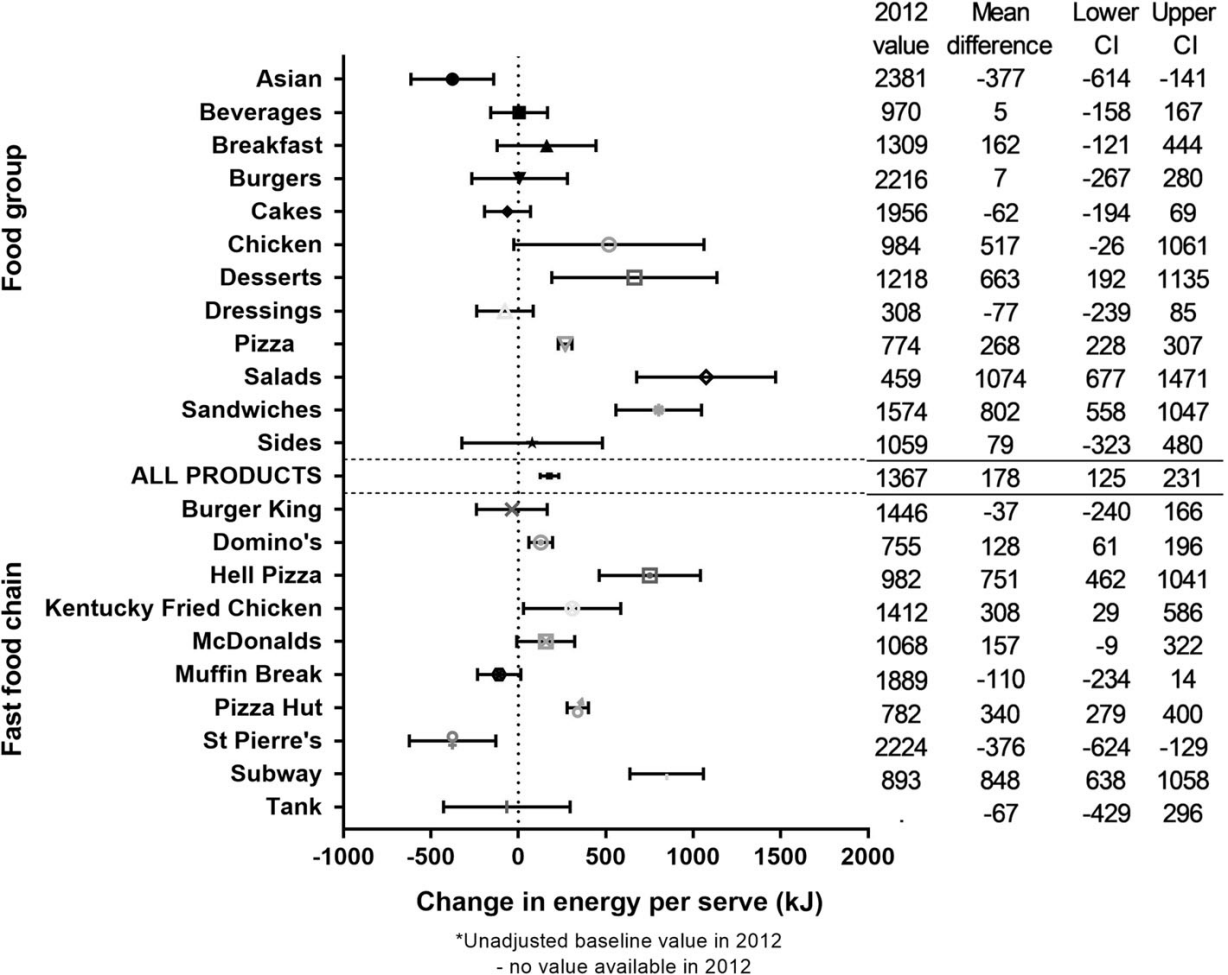
Five year changes in energy per serve of fast food products from major NZ chains (2012 to 2016). * Unadjusted baseline value in 2012. - No value available in 2012

The mean (SD) energy per serve across all products over the 5 years was 1346 (803) kJ, and there was a significant increase over the 5 years of 178 (125 to 231) kJ or + 13% (Fig. 4). Significant interaction was found between year and food group, and between year and fast food chain (*p* < 0.0001). Large, significant increases in energy per serve were observed for four food groups (Desserts, Pizza, Salads, and Sandwiches; + 65, 34, 240, and 61%, respectively) and five fast food chains (Domino’s Pizza, Hell Pizza, Kentucky Fried Chicken, Pizza Hut, and Subway; + 16, 80, 23, 43, and 101%, respectively), and large, significant decreases were observed for one food group (Asian; − 16%), and one fast food chain (St Pierres; − 16%) (Fig. 4).

#### Sodium per serve

**Fig. 5:**
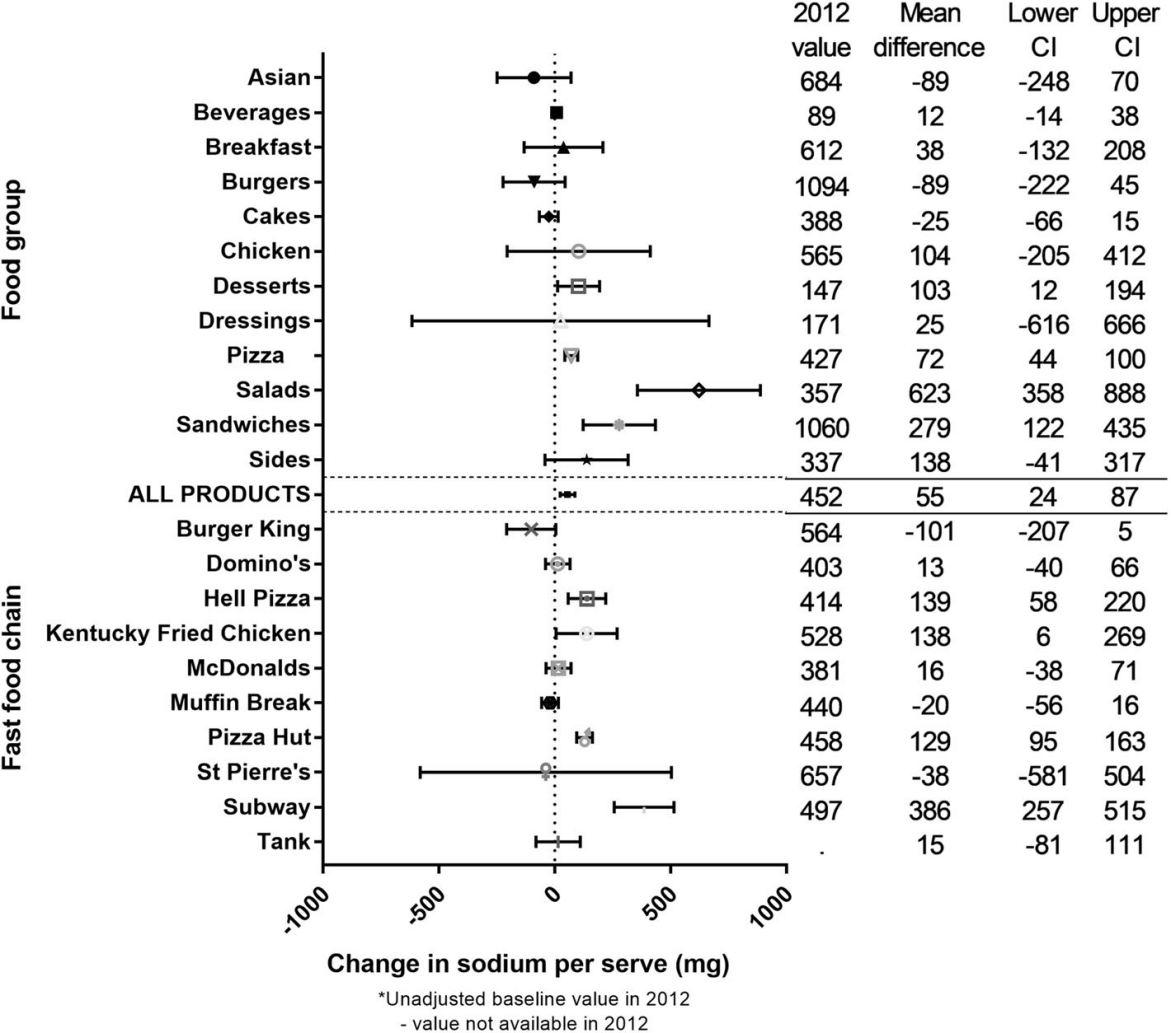
Five year changes in the sodium per serve of fast food products from major NZ chains (2012 to 2016). * Unadjusted baseline value in 2012. - No value available in 2012

The mean (SD) sodium content per serve across all products over the 5 years was 454.0 (458.8) mg, and there was a significant increase over time of + 55.3 (23.9 to 86.7) mg, or + 11% (Fig. 5). Significant interaction was found between year and food group, and between year and fast food chain (*p* < 0.0001). Large, significant increases in sodium per serve were seen for 4/11 food groups (Desserts (+ 92%), Pizza (+ 16%), Salads (+ 176%), and Sandwiches (+ 32%)) and 4/10 fast food chains (Hell Pizza (+ 38%), Kentucky Fried Chicken (+ 24%), Pizza Hut (+ 28%), and Subway (+ 77%)). However, there were no large, significant decreases in sodium per serve for any food group or chain (Fig. 5).

#### Overall findings by food group and fast food chain

Table 1 summarises the overall findings by food group and fast food chain; this table should be interpreted with caution given energy per serve is a function of serve size and energy density, and sodium per serve is a function of serve size and sodium density. Four of 12 food groups displayed large, undesirable changes for the majority of outcomes assessed (≥10%, *p* < 0.05, three or more of five outcomes) i.e. Desserts and Pizza (serve size, energy per serve, and sodium per serve); Sandwiches (energy density, sodium density, energy per serve and sodium per serve); and Salads (sodium density, energy per serve, and sodium per serve). Similarly, four fast food chains displayed large, undesirable changes for the majority of outcomes i.e. Domino’s (serve size, energy density, and energy per serve); Hell Pizza and Pizza Hut (serve size, energy per serve and sodium per serve); and Subway (sodium density, energy per serve, and sodium per serve). Only one food group and one fast food chain displayed large, desirable changes for two or more outcomes i.e. Asian and Subway, where both displayed large significant decreases in serve size and energy per serve.

**Table 1:**
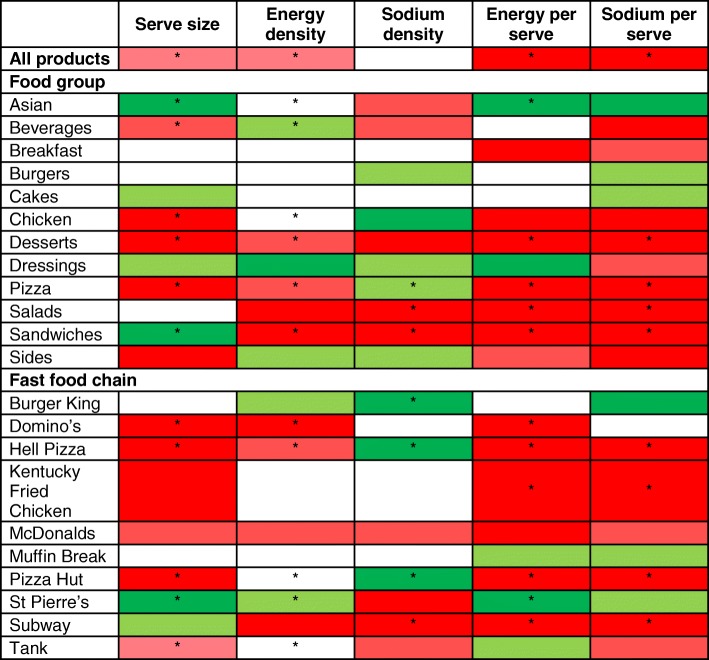
Summary of 5-year changes in the serve size, energy, and sodium content of fast food products sold at major New Zealand chains (2012 to 2016)**

**Dark green indicates ≥10% decrease, light green 9 to 5% decrease, no colour 4% decrease to 4% increase, light red 5 to 9% increase, and dark red ≥10% increase. * Indicates statistically significant change (*p*  < 0.05). Interactions of year with food group and fast food chain with year all had *p* values of <0.001. Table should be interpreted with caution given energy per serve is a function of serve size and energy density, and sodium per serve is a function of serve size and sodium density

### Objective 2: reformulation of products for sale in two or more years

**Fig. 6:**
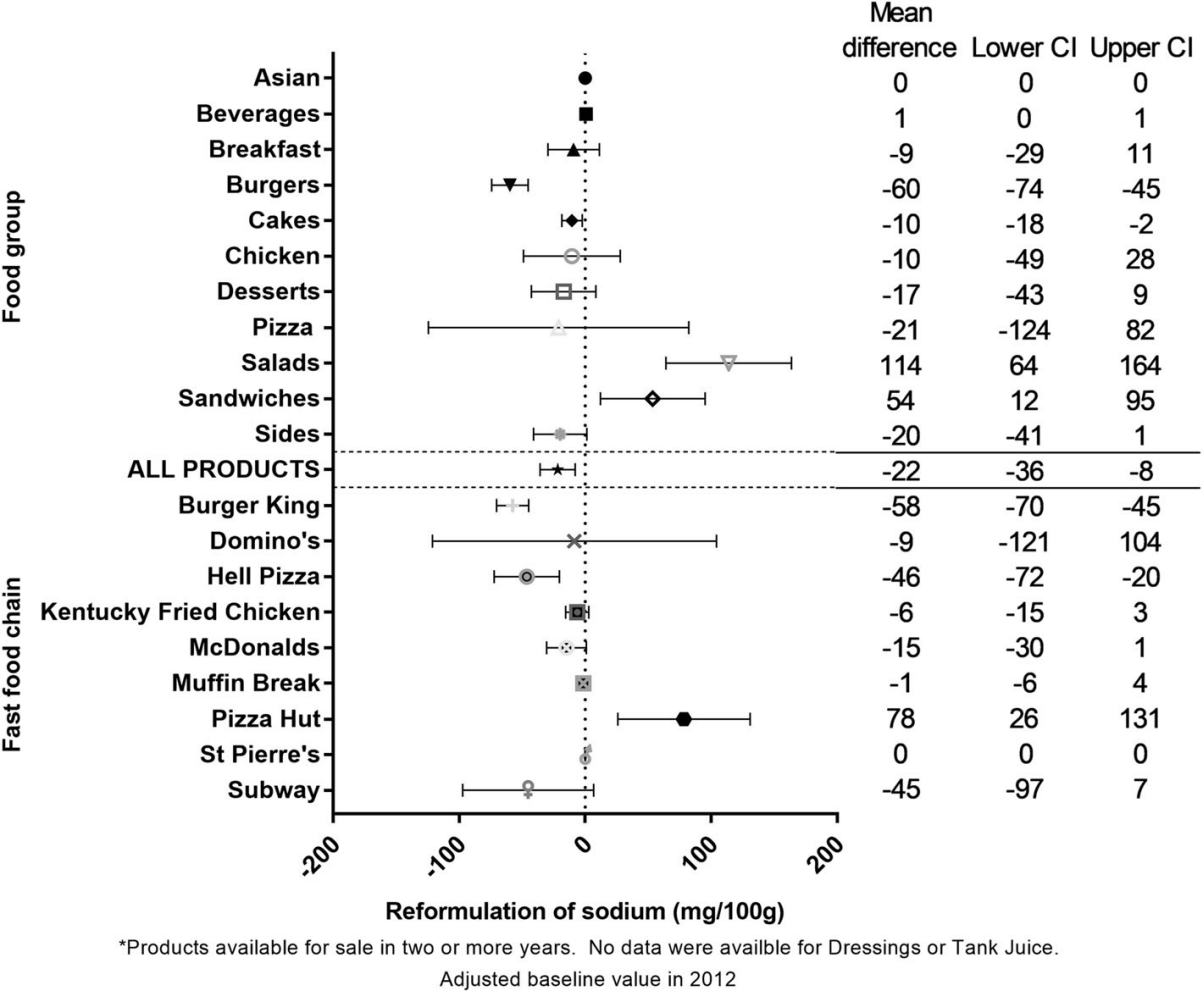
Five year reformulation changes in sodium content of fast food products from major NZ chains (2012 to 2016)*. * Products available for sale in two or more years. No data were available for Dressings or Tank Juice. Adjusted baseline value in 2012

Reformulation analysis on products available for sale in two or more years suggested a moderate and significant reduction over time for sodium density of − 21.8 mg (− 35.7 to − 7.9), or equivalently a 7% reduction. Across the food groups, the largest absolute reduction was seen for Burgers (− 59.7 (− 74.2 to − 45.1) mg/100 g), and across fast food chains the largest absolute reductions were seen for Burger King and Hell Pizza (− 57.5 (− 70.3 to − 44.8) and − 46.2 (− 72.1 to − 20.31) mg/100 g, respectively) (Fig. 6). No reformulation changes were observed for the remaining four outcomes (Additional file 1: Appendix 5).

## Discussion

Overall, we found moderate to large increases in the serve size, energy content (per 100 g and per serve), and sodium content (per serve) of foods and beverages sold at major NZ fast food chains from 2012 to 2016. Of the 12 food groups included, four displayed large (≥10% increase; *p* < 0.05), nutritionally undesirable changes for the majority of (at least three of five) outcomes we assessed i.e. Desserts, Pizza, Sandwiches, and Salads. Similarly, products sold at four of the 10 fast food chains displayed undesirable changes for the majority of outcomes i.e. Domino’s, Hell Pizza, Pizza Hut, and Subway. There were two exceptions, with one food group and one fast food chain making large desirable changes for two or more outcomes i.e. Asian and St Pierre’s (reduced serve size and energy per serve in both cases; Table 1). We found minimal evidence of reformulation of individual products, with no changes in the serve size, energy density, energy per serve, or sodium per serve of products sold in two or more years. The one exception was sodium density where a significant overall reduction of − 7% was observed, largely driven by the Burgers food group, and Burger King and Hell Pizza fast food chains.

A particular strength of this analyses was the comprehensive dataset (*n* = 5468), which means our findings should be broadly generalizable to all fast foods in NZ. However, the limitations include that smaller numbers of products were available for reformulation analyses (*n* = 1025), and matches of same products over time may have been incomplete due to the manual process used and lack of a common identifier. Nonetheless, our findings are robust given we only completed reformulation analyses for sub-groups with >100 products. We also took a cautious approach to reporting sub-group findings by focussing on those food groups with large effect sizes (>10%) and statistical significance (*p* < 0.05). Should the representativeness of products have varied over time e.g. with unhealthy products having a greater probability of being included in later years, then this would generate a selection bias and false trends in our outcome data. However, this is unlikely given we only included food groups and chains with data available each year, and companies are more likely to provide serve size and nutrition information for their full range of products rather than a changing selection. Further, information about fast food purchases are not publicly available in NZ, and thus our data were not sales weighted. However, if there were any bias created we do not think it varied over time, so would not distort trends. Finally, retention of only the largest serve size for duplicate products may have slightly inflated the mean increase in serve size observed over the 5 years. However, this was the case for only 137 products, and retaining the largest serve size in each year means our results reflect the actual, relative increase in all serve sizes available from the included companies.

Trends in the overall nutritional quality of fast foods have also been assessed in Australia and the US. In Australia, the energy density of all fast foods sold at five major chains in New South Wales was assessed over a 7 year period (2009 to 2015) [28]. In contrast to the significant increase we observed across all products over a similar time frame, no change was found in the overall energy content of Australian products, either per 100 g or per serve. However, items on sale for a limited time did increase by 74 kJ per 100 g, which is similar to our finding that the increase in energy density was likely due to new products. In the US Urban and colleagues [29] undertook reformulation analyses and found the energy content per serve of 56% of french fries, cheeseburgers, grilled chicken sandwiches and regular cola (*n* = 27) on sale at three fast food chains increased between 1996 and 2013. Sodium per serve of matched products also increased in 33% of items, despite some reduction in sodium per serve for a small proportion (18%) of products. Our time period was shorter than this previous study (5 years compared with 17), and our data did not include information for all 5 years for all products. However, for 187 products where we had similar, matched outcome data available for 2012 and 2016, we found energy content per serve had increased for 24% of products (and remained unchanged for 46%), and sodium per serve had increased for 24% of products (unchanged for 47%). In another US study, Rudelt et al. [30] examined changes in the sodium density (per 100 g) within 695 lunch and dinner items sold at eight major fast food chains over 14 years (1997/98 to 2009/10), reporting an overall increase of 23% (+ 146 mg/100 g), but with reductions in the sodium content of some side dishes for some chains. We also found a decrease within NZ fast food products due to reformulation of sodium per 100 g; although our drop was smaller (− 7%), we assessed the trend over a shorter (5 years) and more recent (2012 to 2016) time frame.

The overall increases in energy and sodium we observed per serve were attributable to the overall increase in serve size, rather than large increases in energy and sodium concentration per se. For sodium, there were significant reductions per 100 g for Pizza and at Burger King, Hell Pizza, and Pizza Hut, and evidence of reformulation in products available for sale in two or more years. However, these reductions per 100 g did not translate to a drop in sodium per serve, due to the increase in serve size; this is an important finding given larger serve sizes for products such as Beverages and Desserts would result in an increase in total sugar per serve, and for chains such as Domino’s, Hell Pizza, and Pizza Hut, higher saturated fat per serve.

The increases observed in the overall energy and sodium contents of Salads and Sandwiches and Wraps, and across products at Subway and Tank Juice is particularly concerning, as consumers are likely to view these foods and fast food chains as healthier options. Substantial increases in the numbers of products available in these food groups (salads increased from 20 in 2012 to 67 in 2016), and changes in the types of options available (there was a 600% increase in salads including heavy root vegetables and legumes and grains) may account for some of the increase in energy observed, despite no significant change in serve size.

An important consideration of our analyses is that it excluded fish and chips, which are an iconic part of the NZ fast food culture; in 2017 42% of NZ adults consumed fish and chips in the past month [7]. However, fish and chip outlets are independently owned and operated, and while there are important opportunities to improve the type of fat [31] and amount of added salt, the operating structure of fast food restaurants within chains provides substantial and widespread opportunities for reformulation. Further, the consumption of products from fast food companies in NZ is likely to be much larger overall than that of fish and chips, with McDonalds and Kentucky Fried Chicken alone being consumed by 38 and 23% of NZ adults respectively, in the last month [7]. Growth over the past 2 years was also higher for many fast food chains (39% for Domino’s Pizza, 30% for Sushi, and 20% for Indian) compared with fish and chips (11%).

Another important consideration is the impact our findings might have on children and young people, the highest consumers of fast foods [32]. Products on sale at McDonalds, the one fast food chain which made a ‘Healthy Kids Industry Pledge’ [23] displayed no overall changes in serve size or nutrient content. A separate analysis of products included in children’s Happy Meals was similar with no overall changes in product profiles or evidence of reformulation [33].

## Conclusions

In conclusion, NZ fast foods have become larger and more energy dense over the past 5 years. Lower sodium concentration in new and reformulated products has been offset by overall increases in serve size. Systematic monitoring and implementation of Government-led targets for serve size, energy and nutrient content of fast food products could improve the composition of NZ fast foods and population diets; if improvements are not observed then regulation should be considered. Products marketed to and favoured by children and young people should also be carefully considered, because they are the most frequent consumers of fast food. Finally, in NZ, fast food chains should be encouraged to make ‘Healthy Kids Industry’ pledges [23] outlining measurable improvements to the serve size, energy, and nutrient content of their products.

## Additional file

Additional file 1: **Appendix 1.** Preparation of fast food data for analysis. **Appendix 2.** Total products and unadjusted mean (SD) values included in main analyses by year, food group, fast food chain, and outcome*. **Appendix 3.** Summary of products included in reformulation analyses sold in two or more years by year, food group, fast food chain, and outcome. **Appendix 4.** Model adjusted means, 95% CI’s and *p*-values for all products in main analyses by year, food group, and outcome*. **Appendix 5.** Model adjusted means, 95% CI’s and *p*-values for reformulation analyses including products available for sale in two or more years by year, fast food chain, and outcome*. (XLSX 134 kb)
